# Supplementary material for: CO2 hydrogenation over Fe-Co bimetallic catalysts with tunable selectivity through a graphene fencing approach
Source: Nat Commun. 2024 Jan 13;15:512. doi: 10.1038/s41467-024-44763-9 (PMC10787759; doi:10.1038/s41467-024-44763-9)
Supplement: Supplementary file 1 — Supplementary Information [file 41467_2024_44763_MOESM1_ESM.pdf]

Supporting Information File for

CO_2 hydrogenation over Fe-Co bimetallic catalysts with tunable selectivity through a graphene fencing approach

Jiaming Liang [1,#], Jiangtao Liu [2,#], Lisheng Guo [3]*, Wenhang Wang [1], Chengwei Wang [1], Weizhe Gao [1], Xiaoyu Guo [1], Yingluo He [1], Guohui Yang [1], Shuhei Yasuda [1]*, Bing Liang [2]*, Noritatsu Tsubaki [1]*

[1] Department of Applied Chemistry, School of Engineering, University of Toyama, Gofuku 3190, Toyama 930-8555, Japan

[2] School of Materials Science and Engineering, Shenyang University of Chemical Technology, Shenyang, Liaoning 110142, China

[3] School of Chemistry and Chemical Engineering, Anhui University, Hefei, Anhui 230061, China

[#] These authors contributed equally: Jiaming Liang, Jiangtao Liu.

Corresponding authors: tsubaki@eng.u-toyama.ac.jp (N. Tsubaki), lsguo@ahu.edu.cn (L. Guo), liangbing@syuct.edu.cn (B. Liang), yasu@eng.u-toyama.ac.jp (S. Yasuda)

Supplementary Methods

Calculation methods for conversion and product selectivity. The CO_2 conversion, CO selectivity, and hydrocarbon selectivity were calculated using equations 1, 2, and 3, respectively.

$$CO_2 \text{ conversion (\%)} = (CO_{2-in} - CO_{2-out}) / CO_{2-in} \times 100\% \quad (1)$$

CO_2-in and CO_2-out represented the mole fractions of CO_2 in the intake and exit.

$$\text{CO selectivity (\%)} = CO_{-out} / (CO_{2-in} - CO_{2-out}) \times 100\% \quad (2)$$

Where CO_{-out} symbolized the mole fraction of CO in the outlet.

$$C_i \text{ hydrocarbon selectivity } (C_{-mol}\ \%) =$$

$$(\text{Moles } of \text{ Ci hydrocarbons } \times i) / (\textstyle\sum_{i=1}^{n} \text{moles } of \text{ Ci hydrocarbons } \times i) \times 100\% \quad (3)$$

Theoretical calculations method. We have employed the Vienna Ab Initio Package (VASP) to perform all the spin-polarized density functional theory (DFT) calculations within the generalized gradient approximation (GGA) using the PBE formulation [1-3]. We have chosen the projected augmented wave (PAW) potentials to describe the ionic cores and take valence electrons into account using a plane wave basis set with a kinetic energy cutoff of 400 eV [4-5]. We allowed partial occupancies of the Kohn−Sham orbitals using the Gaussian smearing method with a width of 0.05 eV. The electronic energy was considered self-consistent when the energy change was smaller than 5−10 eV, and a geometry optimization was considered convergent when the force change was smaller than 0.02 eV/Å. Grimme's DFT-D3 methodology was used to describe the dispersion interactions [6].

The equilibrium lattice constants of monoclinic Fe_5C_2 unit cell were optimized, when using a 2×6×6 Monkhorst-Pack k-point grid for Brillouin zone sampling; the resulting lattice to be a=11.676 Å, b=4.796 Å, c=5.137 Å, α=90°, β=100.8°, γ=90°. We then used it to construct a Fe_5C_2(510) surface model (model 1) with p (2×1) periodicity in the x and y directions and 3

stoichiometric layers in the z direction separated by a vacuum layer in the depth of 15 Å in order to separate the surface slab from its periodic duplicates. Model 1 comprised of 60 Fe and 24 O atoms. Model 2 was built by adding one Co_{10} cluster onto model 1, and Model 3 was built by adding one Co_8C_4 cluster onto model 1. For structural optimizations, a 2×2×1 k-point grid in the Brillouin zone was used for k-point sampling, and the bottom two stoichiometric layers were fixed while the top one was allowed to relax.

The adsorption energy (Eads) of adsorbate A was defined as $E_{ads} = E_{A/surf} - E_{surf} - E_{A\,(g)}$, where $E_{A/surf}$, E_{surf} and E_A (g) are the energies of adsorbate A adsorbed on the surface, the energy of clean surface, and the energy of isolated A molecule in a cubic periodic box with a side length of 20 Å and a 1×1×1 Monkhorst-Pack k-point grid for Brillouin zone sampling, respectively.

Four layers of model 1 and model 2 were employed to simulate the catalysis of the reduction of C_3H_6. Atoms in the upper two layers of the surface were allowed to move freely while the bottom two layers of surface were fixed to simulate the surface of structure. The Monkhorst-Pack-grid-mesh-based Brillouin zone k-points were set as 2×2×1 for all periodic structure with the cutoff energy of 400 eV. The convergence criteria were set as 0.01 eV A^{-1} and 10^{-5} eV in force and energy, respectively.

The free energy calculation of species adsorption (ΔG) is based on following model.

$$\Delta G = \Delta E + \Delta E_{ZPE} + \Delta H_{0-T} - T\Delta S$$

Herein, ΔE, ΔE_{ZPE}, and ΔS respectively represent the changes of electronic energy, zero-point energy, and entropy that caused by adsorption of intermediate. The $\Delta H_0 \rightarrow T$ refers to the change in enthalpy when heating from 0 K to T K.

The climbing image nudged elastic band (CI-NEB)[5] was employed with converged force less than 0.05 eV/Å in VTST[6] package to obtain transition states as a function of applied potential

to derive free energy barriers.

Supplementary Figures

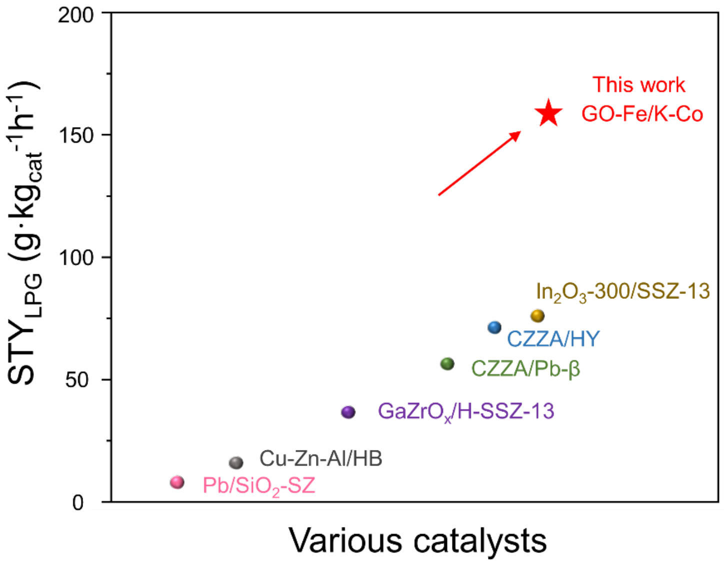

Supplementary Figure 1 | STY$_{LPG}$ of GO-Fe/K-Co compared with other catalysts. Catalysts from left to right were cited from the references [7–12].

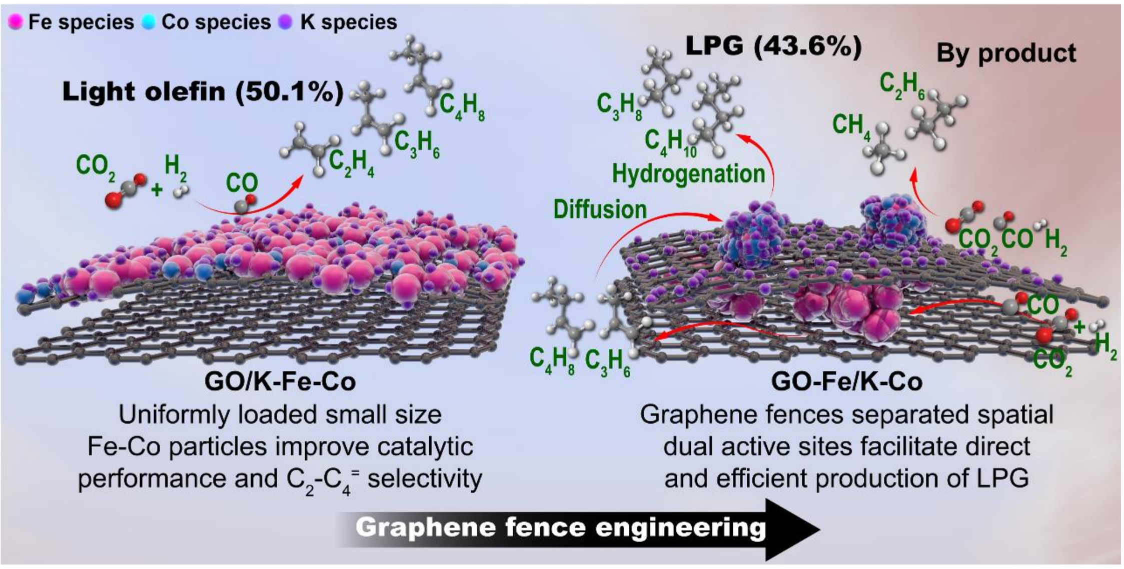

Supplementary Figure 2 | Schematic diagram of switching product types by Fe-Co active sites with various distributions controlled by graphene fences. Fe species, red. Co species, blue. K species, purple.

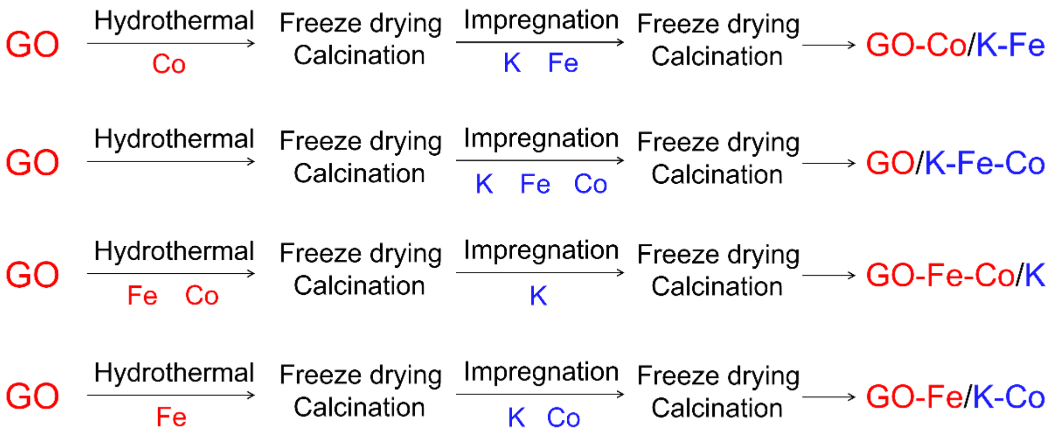

Supplementary Figure 3 | Synthesis of Fe-Co bimetallic catalysts with different distributions.

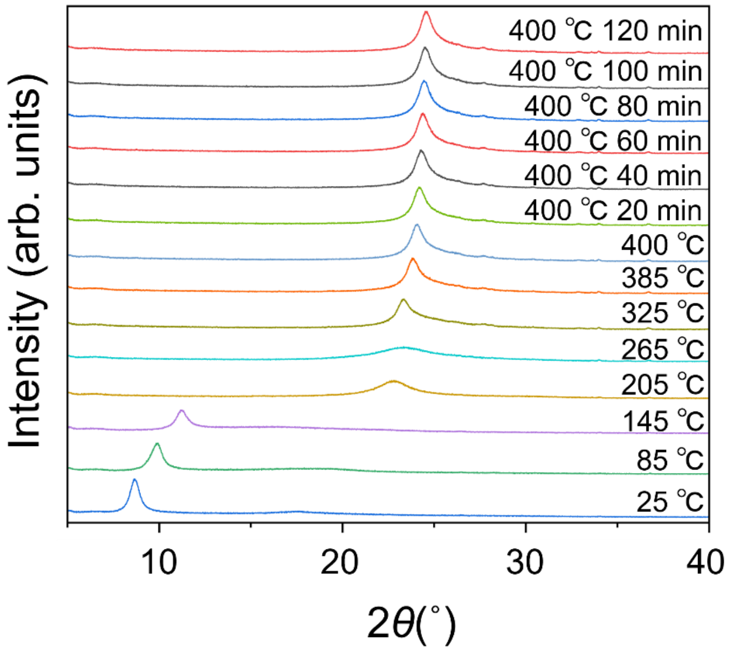

Supplementary Figure 4 | *In situ* XRD for GO during temperature programmed reduction.

Pure H_2, 30 mL/min, 25–400 °C, 3 °C/min, atmospheric pressure.

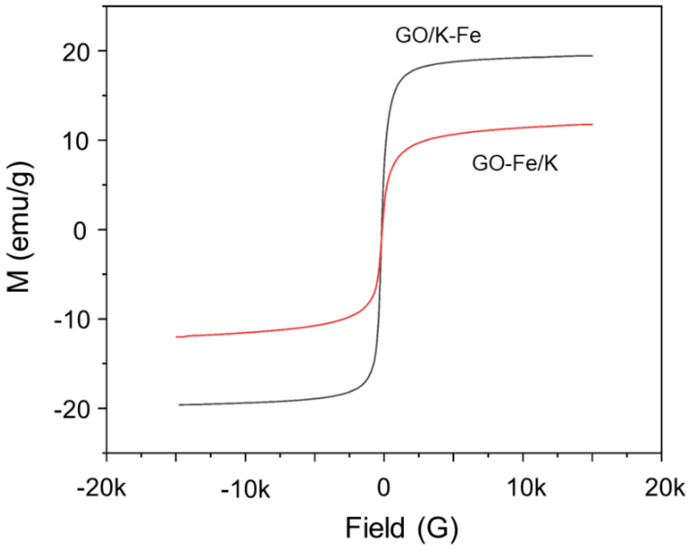

Supplementary Figure 5 | Room temperature M-H loop of GO/K-Fe and GO-Fe/K.

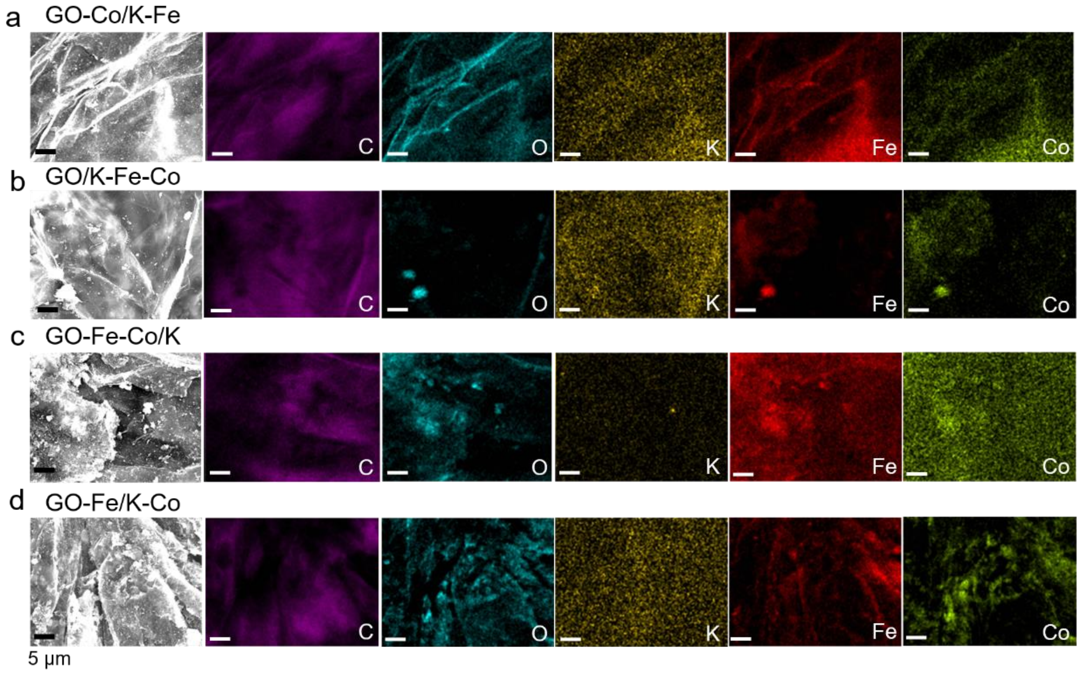

Supplementary Figure 6 | SEM mapping images of graphene-supported Fe-Co catalysts.

(**a**) GO-Co/K-Fe, (**b**) GO/K-Fe-Co, (**c**) GO-Fe-Co/K, and (**d**) GO-Fe/K-Co. The bars stand for 5 μm.

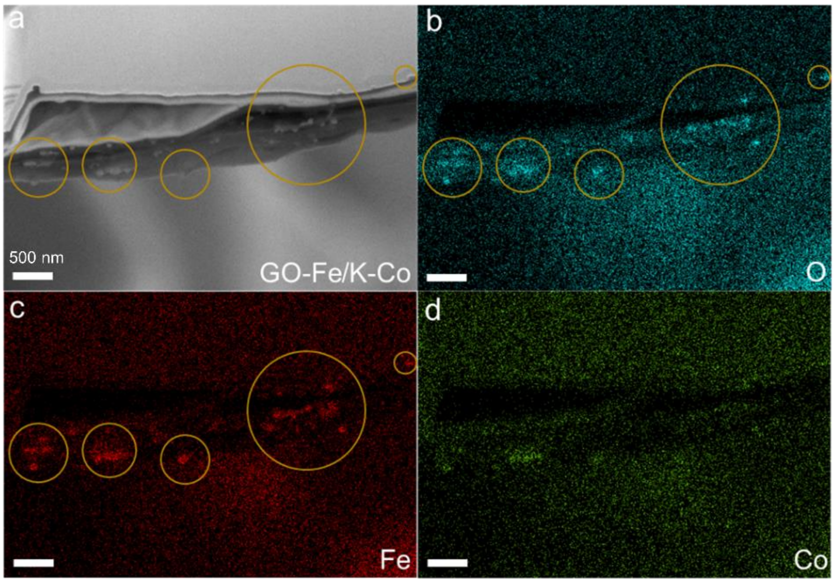

Supplementary Figure 7 | Cross-section and mapping elemental images of GO-Fe/K-Co obtained by FIB-SEM. (**a**) Cross-section SEM images of GO-Fe/K-Co, (**b**) Elemental mapping images of O, (**c**) Elemental mapping images of Fe, and (**d**) Elemental mapping images of Co. The bars stand for 500 nm.

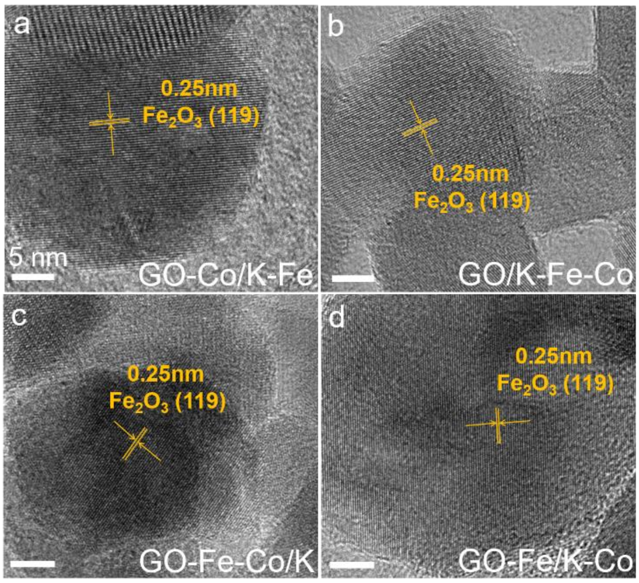

Supplementary Figure 8 | HR-TEM images of the fresh graphene-supported Fe-Co catalysts. (**a**) GO-Co/K-Fe, (**b**) GO/K-Fe-Co, (**c**) GO-Fe-Co/K, and (**d**) GO-Fe/K-Co. The bars stand for 5 nm.

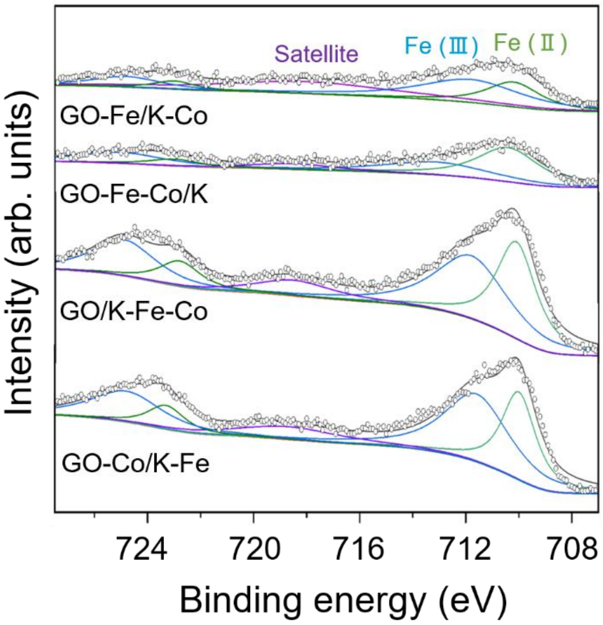

Supplementary Figure 9 | Fe 2*p* XPS spectra of fresh graphene-supported Fe-Co catalysts.

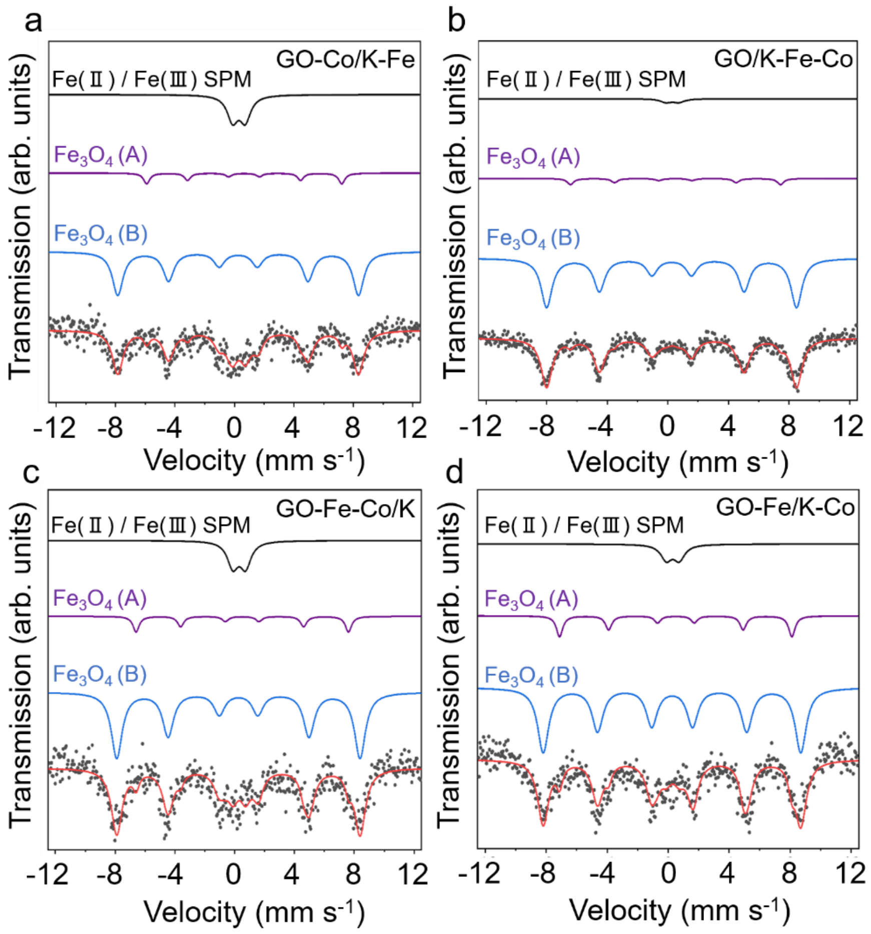

Supplementary Figure 10 | ^{57}Fe Mössbauer spectra of the fresh graphene-supported Fe-Co catalysts. (**a**) GO-Co/K-Fe, (**b**) GO/K-Fe-Co, (**c**) GO-Fe-Co/K, and (**d**) GO-Fe/K-Co.

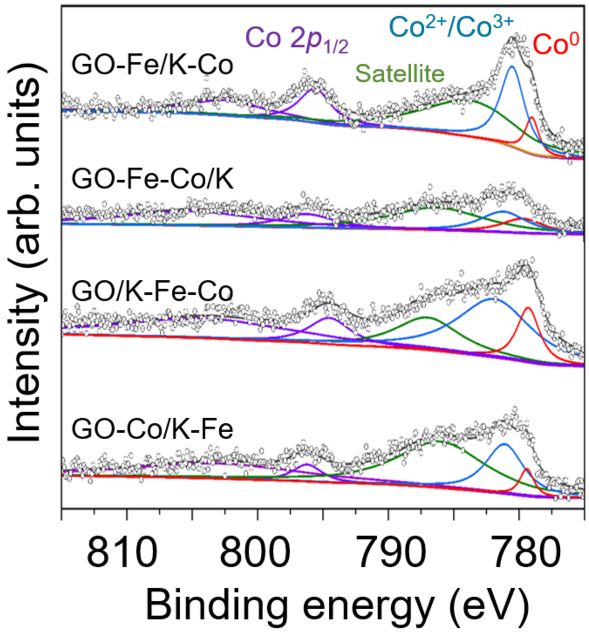

Supplementary Figure 11 | Co 2*p* XPS spectra of fresh graphene-supported Fe-Co catalysts.

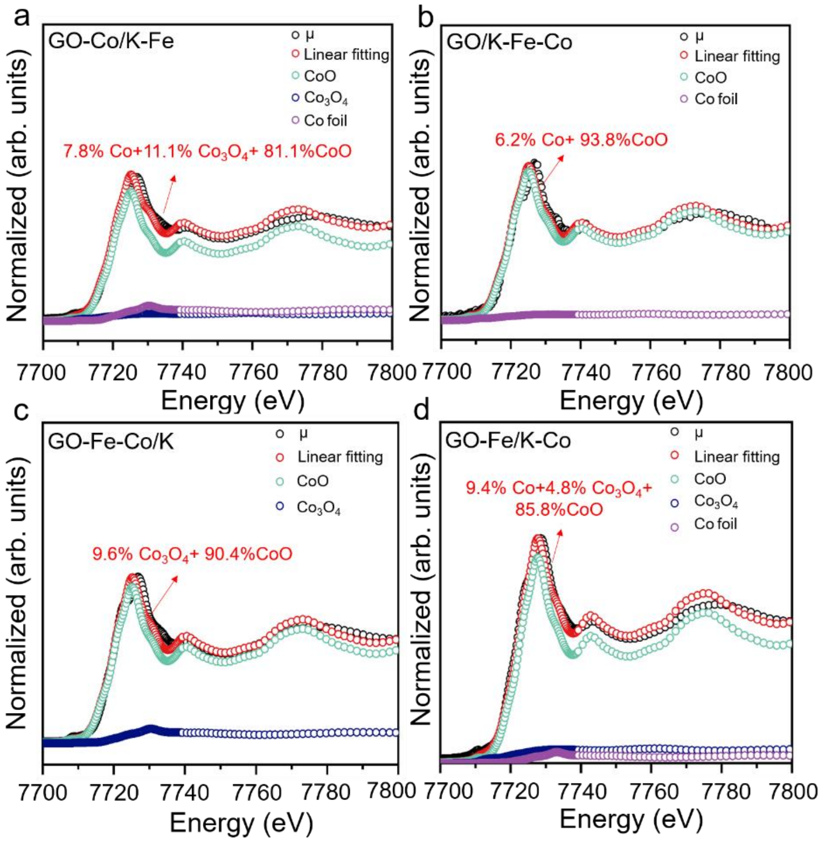

Supplementary Figure 12 | XANES spectra with fitting curves of the fresh graphene-supported Fe-Co catalysts. (**a**) GO-Co/K-Fe, (**b**) GO/K-Fe-Co, (**c**) GO-Fe-Co/K, and (**d**) GO-Fe/K-Co.

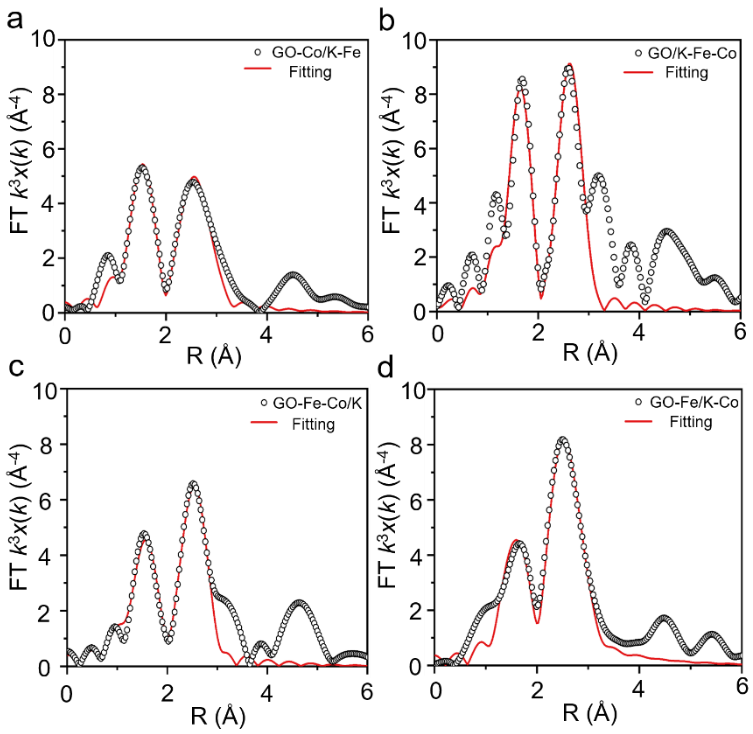

Supplementary Figure 13 | The EXAFS fitting results of the fresh graphene-supported Fe-Co catalysts. (**a**) GO-Co/K-Fe, (**b**) GO/K-Fe-Co, (**c**) GO-Fe-Co/K, and (**d**) GO-Fe/K-Co.

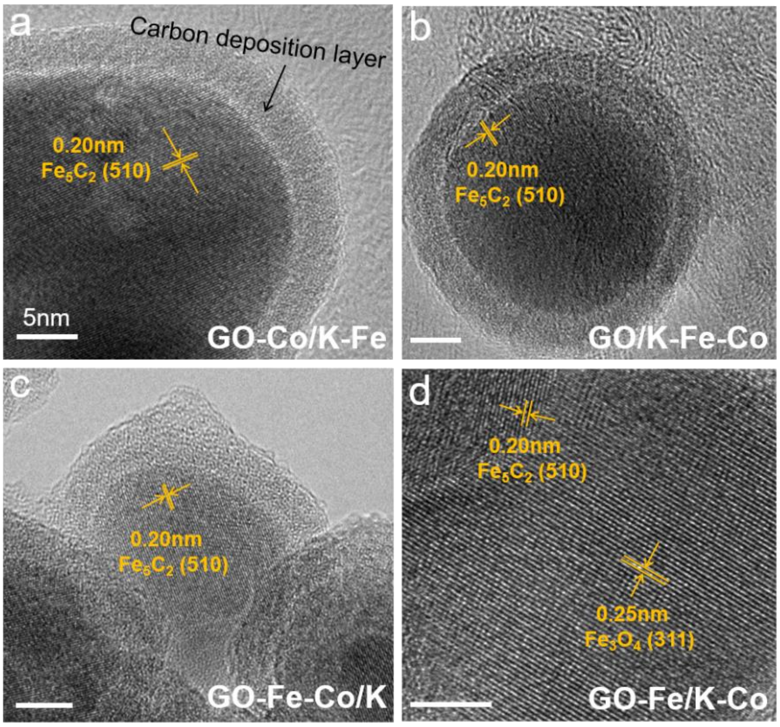

Supplementary Figure 14 | HR-TEM images of the spent graphene-supported Fe-Co catalysts. (**a**) GO-Co/K-Fe, (**b**) GO/K-Fe-Co, (**c**) GO-Fe-Co/K, and (**d**) GO-Fe/K-Co. The bars stand for 5 nm.

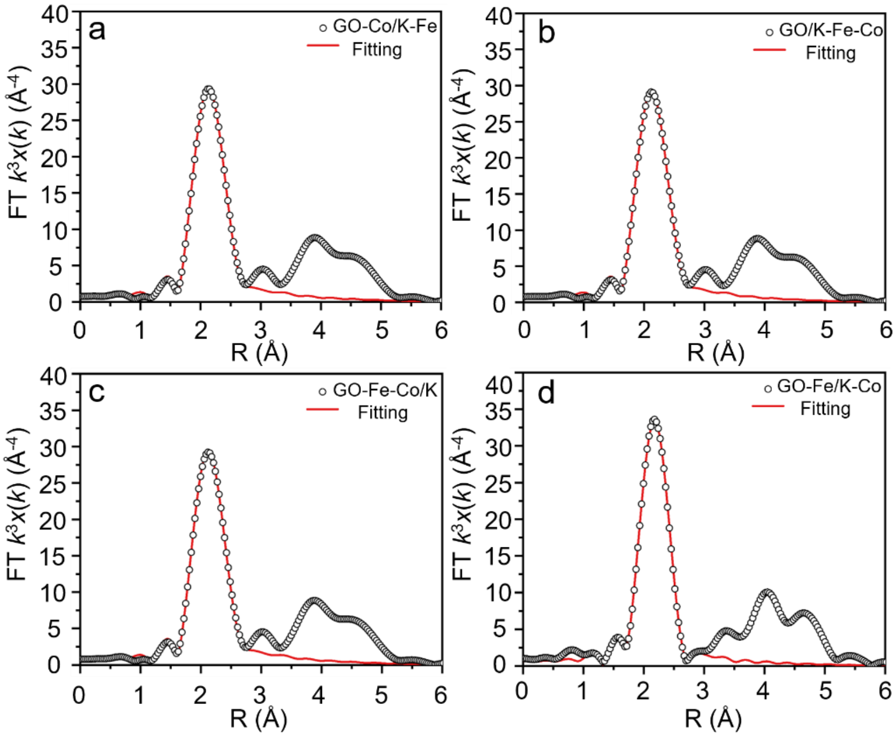

Supplementary Figure 15 | The EXAFS fitting results of the spent graphene-supported Fe-Co catalysts. (**a**) GO-Co/K-Fe, (**b**) GO/K-Fe-Co, (**c**) GO-Fe-Co/K, and (**d**) GO-Fe/K-Co.

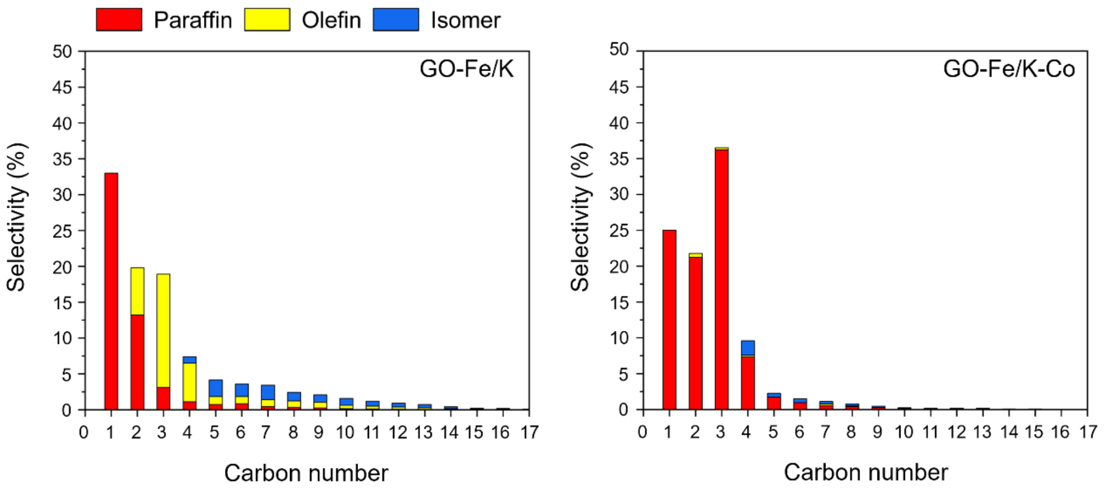

Supplementary Figure 16 | Detailed product distributions of GO-Fe/K and GO-Fe/K-Co catalysts.

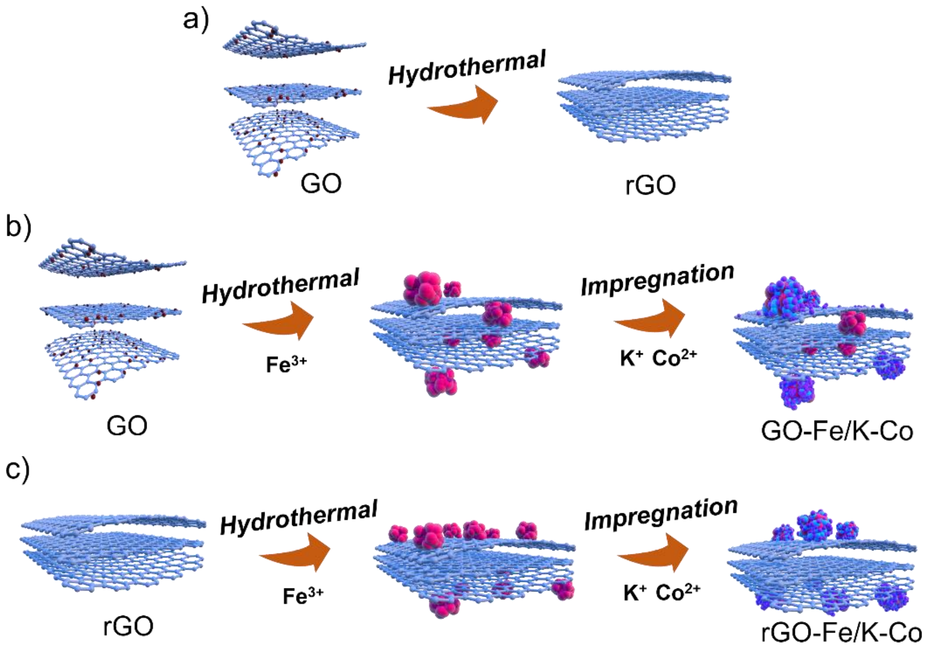

Supplementary Figure 17 | Preparation processes and metal distributions of GO-Fe/K-Co and rGO-Fe/K-Co. (**a**), synthesis of rGO; (**b**), synthesis of GO-Fe/K-Co; (**c**), synthesis of rGO-Fe/K-Co.

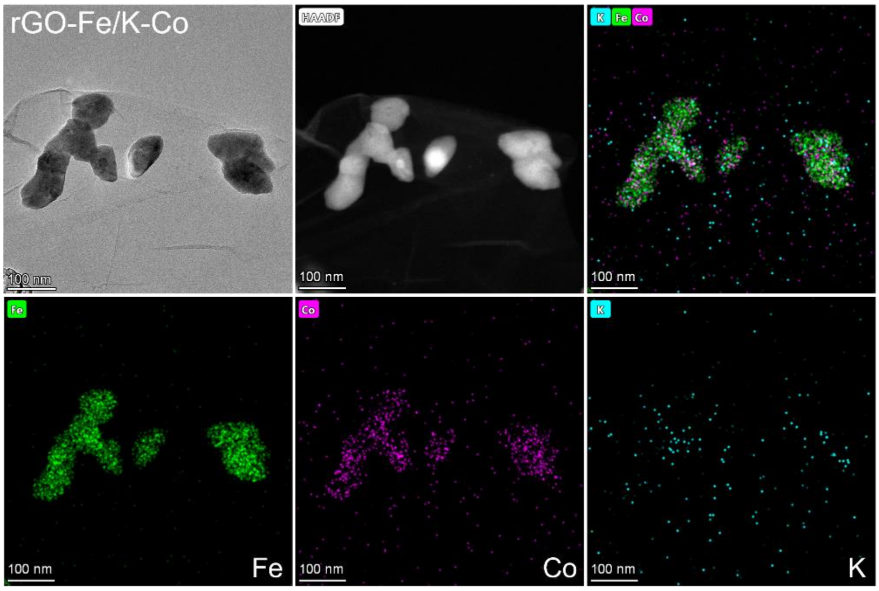

Supplementary Figure 18 | The TEM elemental mapping images of rGO-Fe/K-Co. The bars stand for 100 nm.

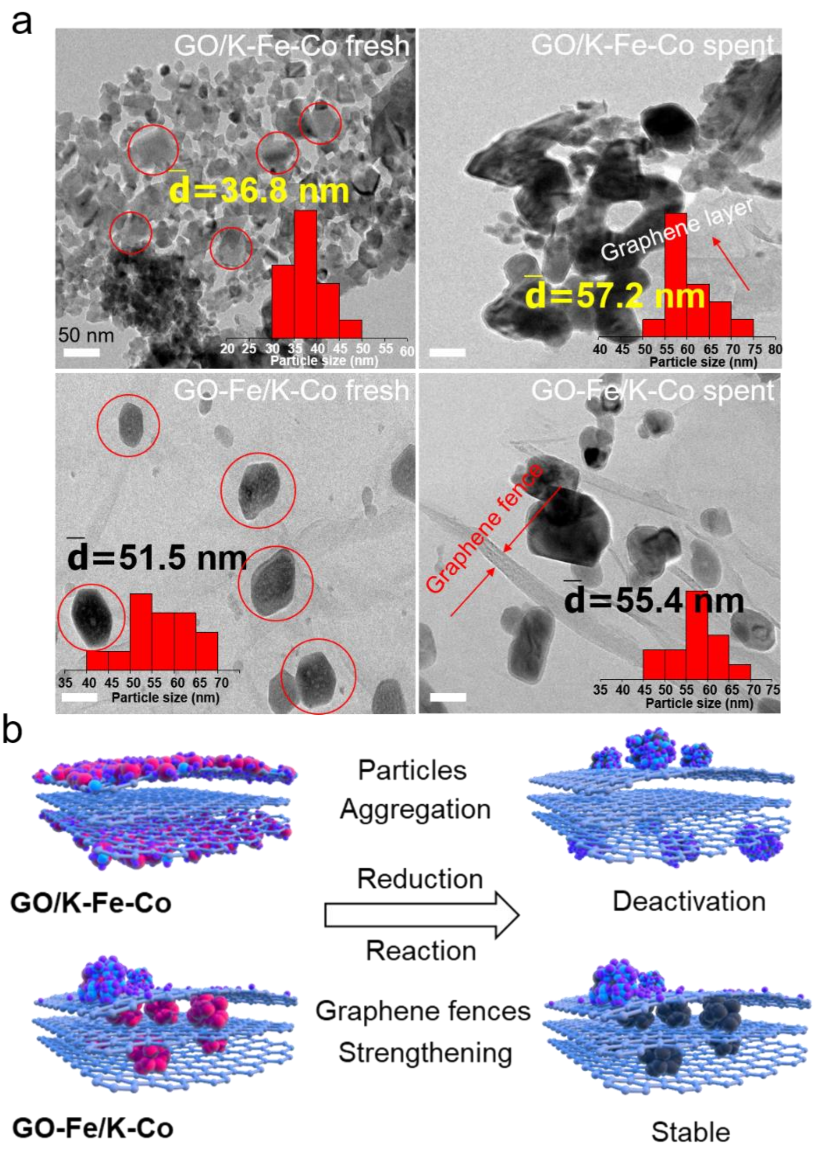

Supplementary Figure 19 | Particle sizes of the metal and schematic diagram of the protection effect of graphene fences. (**a**) The particle size of GO/K-Fe-Co and GO-Fe/K-Co catalysts**;** the bars stand for 50 nm. (**b**) Schematic diagram of graphene fences protect metal particle from deactivation. Fresh Fe species, red; spent Fe species, black; Co, blue; K, purple.

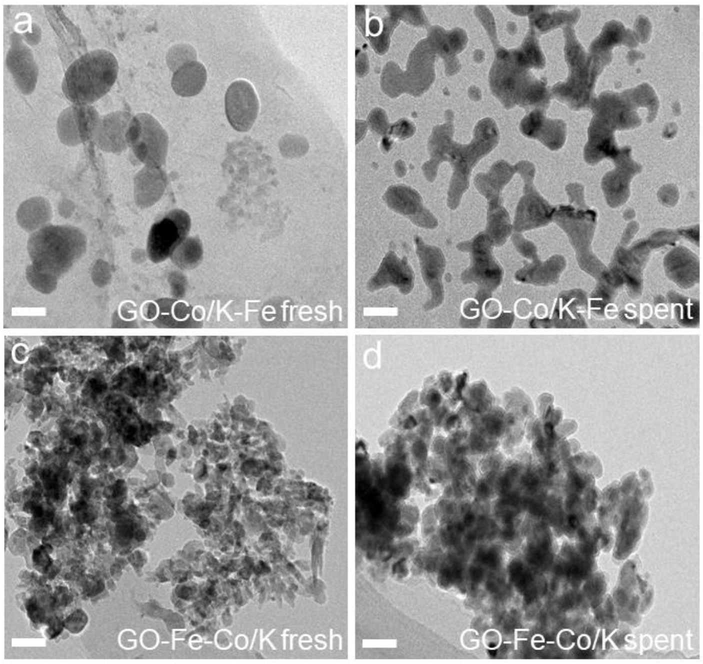

Supplementary Figure 20 | TEM images of fresh and spent GO-Co/K-Fe and GO-Fe-Co/K catalysts. (**a**) GO-Co/K-Fe fresh catalyst, (**b**) GO-Co/K-Fe spent catalyst, (**c**) GO-Fe-Co/K fresh catalyst, and (**d**) GO-Fe-Co/K spent catalyst. The bars stand for 50 nm.

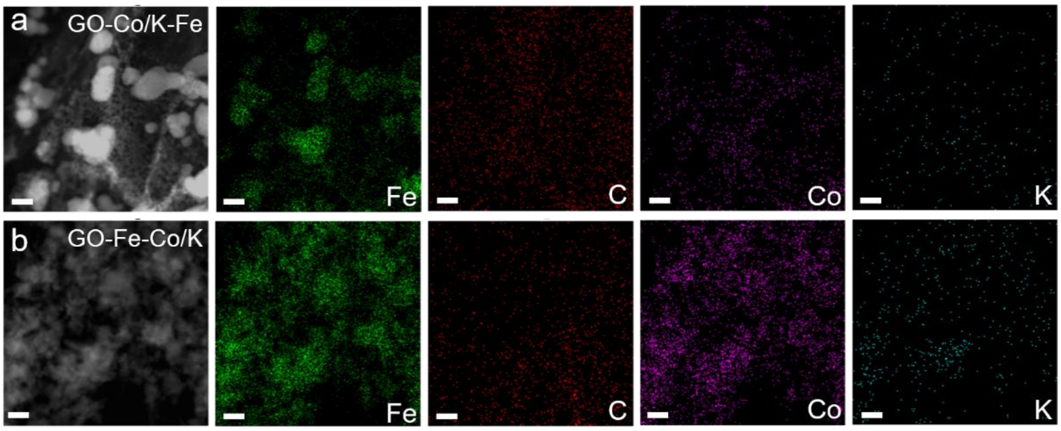

Supplementary Figure 21 | TEM mapping images of fresh GO-Co/K-Fe and GO-Fe-Co/K catalysts. (**a**) GO-Co/K-Fe, and (**b**) GO-Fe-Co/K. The bars stand for 50 nm.

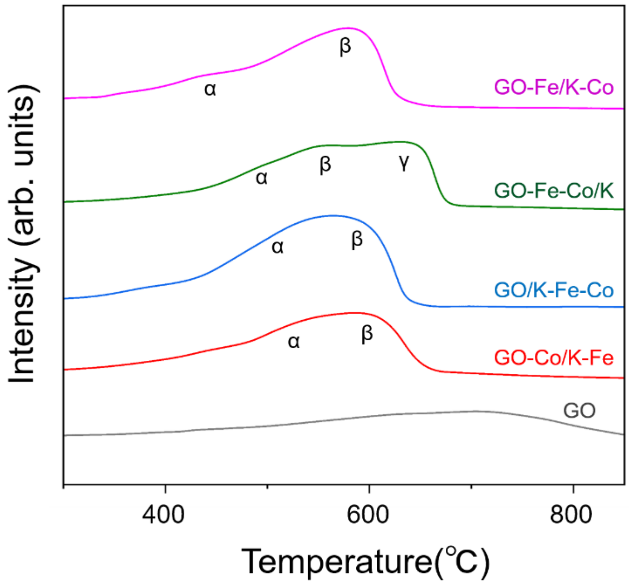

Supplementary Figure 22 | H_2-TPR profile of the catalysts.

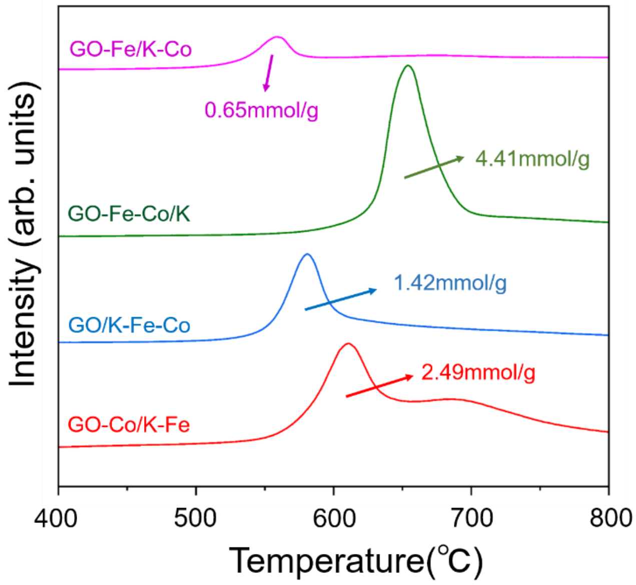

Supplementary Figure 23 | CO_2-TPD profile of the catalysts.

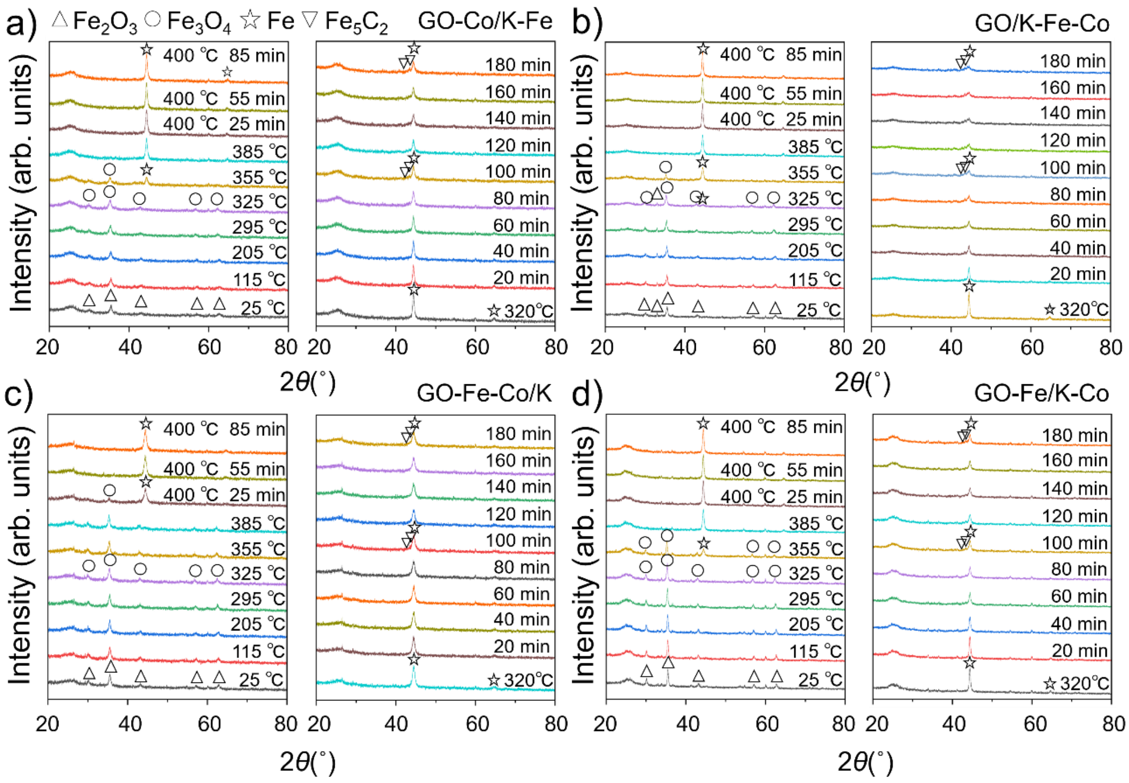

Supplementary Figure 24 | *In situ* XRD patterns for the reduction and carbonization processes of catalysts. (**a**) GO-Co/K-Fe, (**b**) GO/K-Fe-Co, (**c**) GO-Fe-Co/K, and (**d**) GO-Fe/K-Co. The left images in each catalyst represent the reduction process in a hydrogen atmosphere, and the right images in each catalyst represent the carbonization process in a reactant gas atmosphere. Test conditions: Pure H_2 from 25–400 °C and reactant gas (CO_2/H_2) at 320 °C, atmospheric pressure, 30 mL/min.

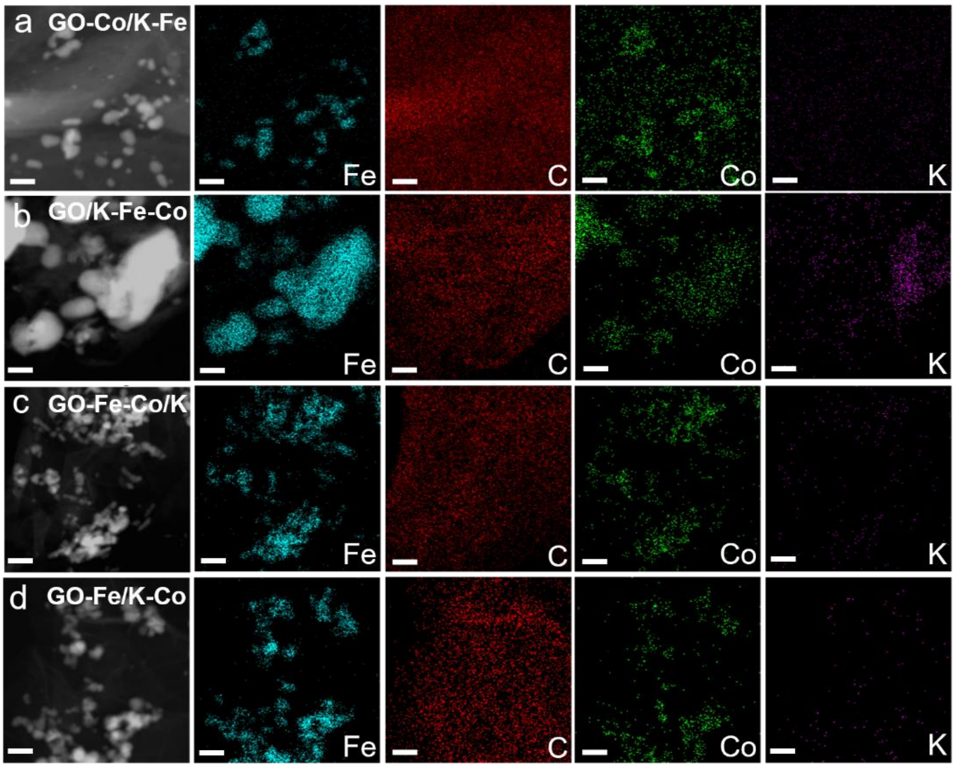

Supplementary Figure 25 | TEM mapping images of spent catalysts. (**a**) GO-Co/K-Fe, (**b**) GO/K-Fe-Co, (**c**) GO-Fe-Co/K, and (**d**) GO-Fe/K-Co. The bars stand for 50 nm.

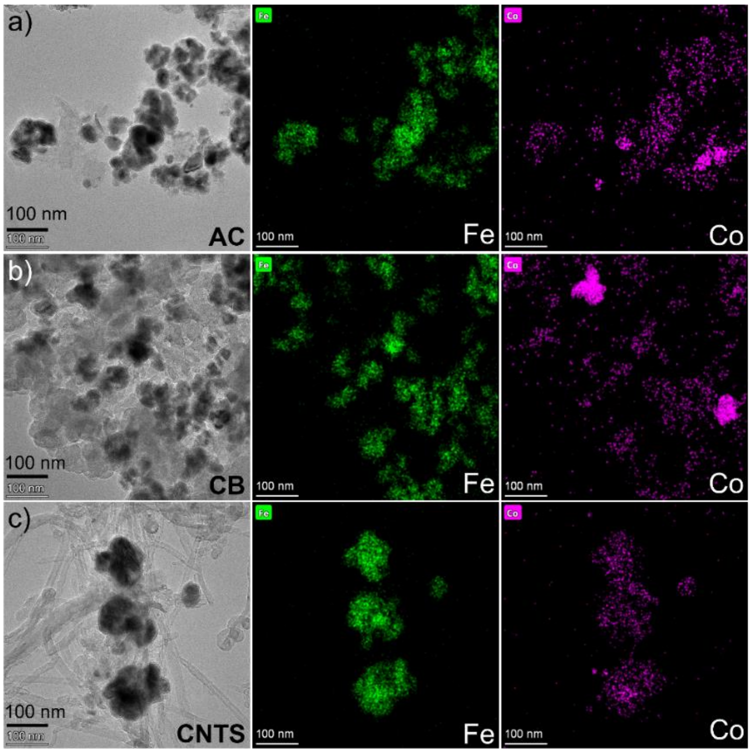

Supplementary Figure 26 | TEM elemental mapping distributions of Fe and Co. (a) AC-Fe/K-Co. (b) CB-Fe/K-Co. (c) CNTS-Fe/K-Co. The bars stand for 100 nm.

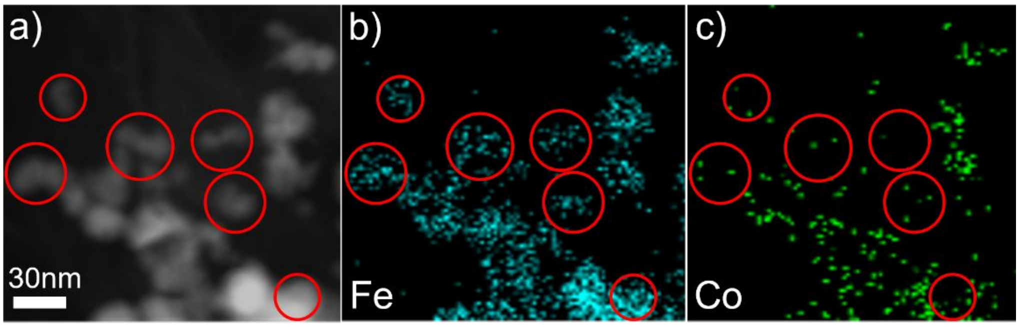

Supplementary Figure 27 | TEM and mapping elemental images of spent GO-Fe/K-Co. The bars stand for 30 nm. (**a**) TEM images of spent GO-Fe/K-Co catalyst, (**b**) TEM elemental mapping images of Fe, and (**c**) TEM elemental mapping images of Co. The red circles represent the area with different Fe and Co distributions.

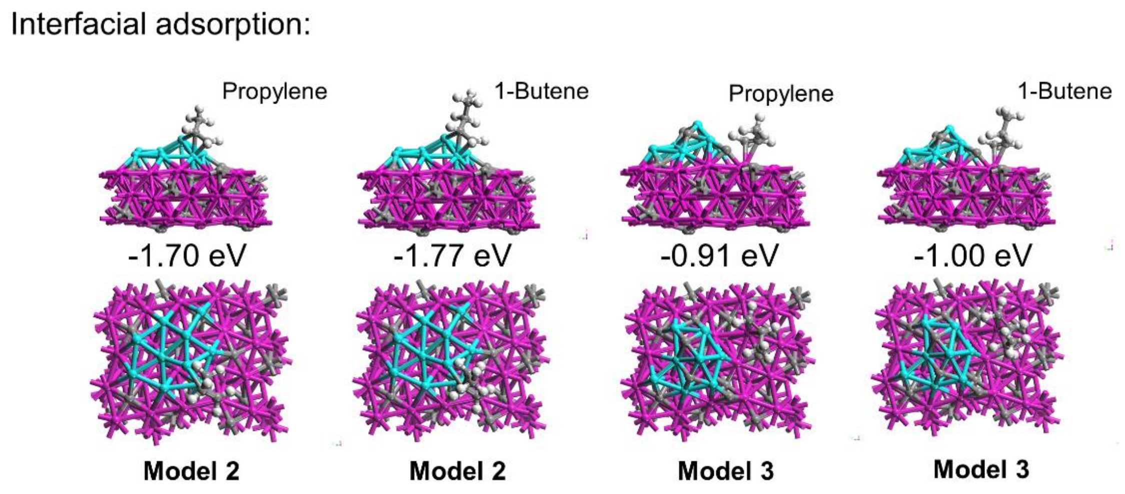

Supplementary Figure 28 | Interfacial adsorption models and adsorption energy. Substrate colors: Fe, pink; C, brown; Co, blue; O, white.

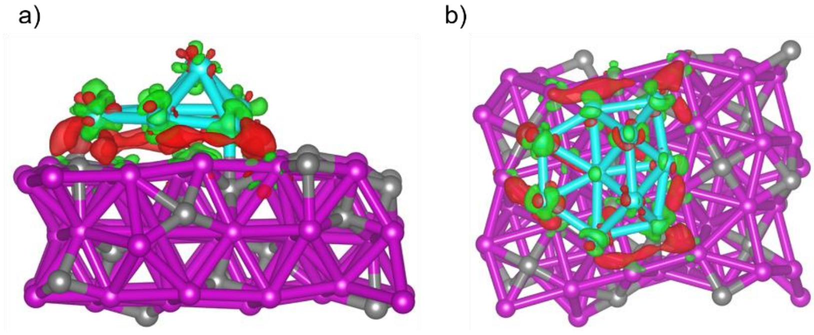

Supplementary Figure 29 | Charge density difference of Model 2. (**a**) Side view, and (**b**) Top view. The red and green colors represented electron accumulation and loss, respectively.

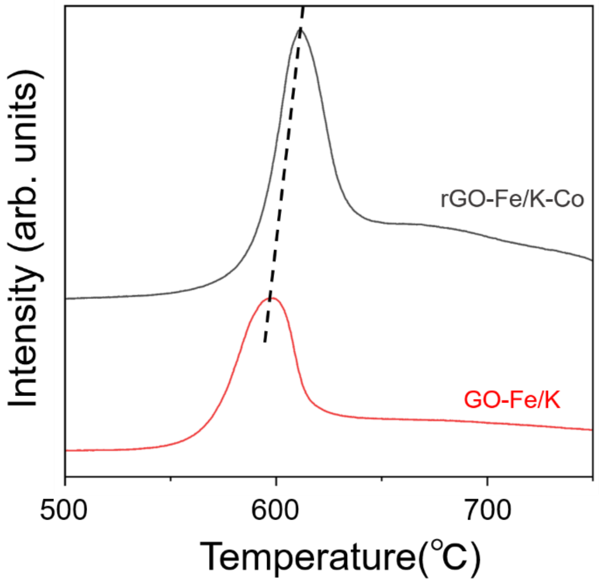

Supplementary Figure 30 | H_2-TPD profile of rGO-Fe/K-Co and GO-Fe/K.

Note: The unitary peak of GO-Fe/K shifted to a lower temperature compared with rGO-Fe/K-Co, providing compelling evidence that the addition of Co enhanced the hydrogen adsorption strength.

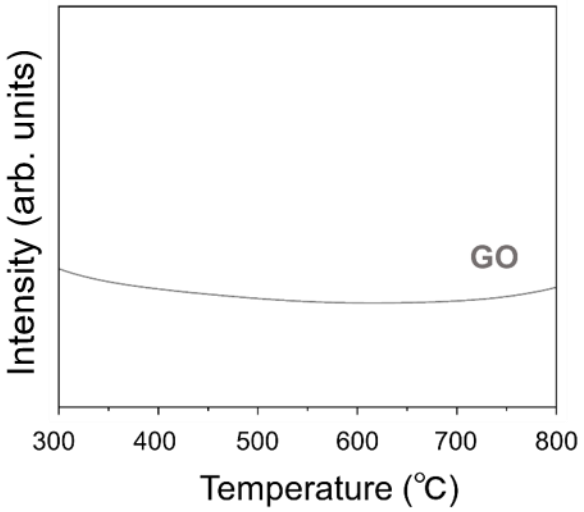

Supplementary Figure 31 | H_2-TPD profile of GO.

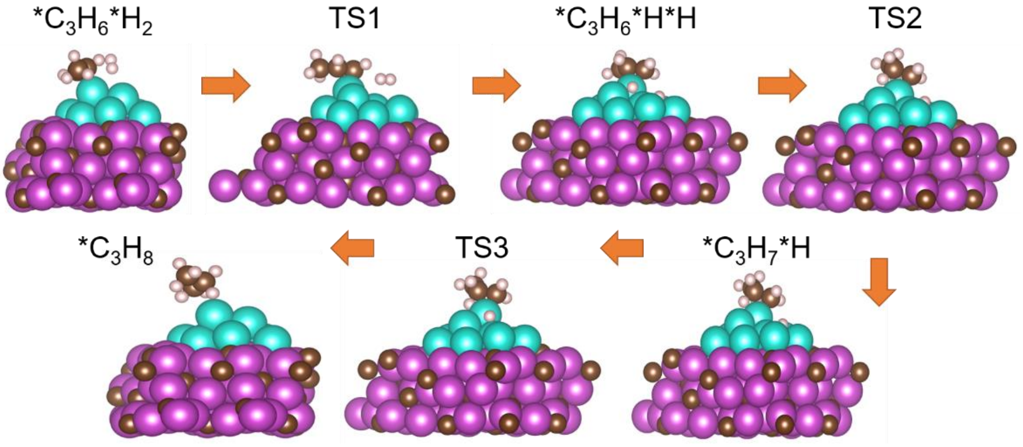

Supplementary Figure 32 | Side-view of propylene hydrogenation reaction coordinate over Fe_5C_2-Co site. Substrate colors: Fe, pink; C, brown; Co, blue; O, white.

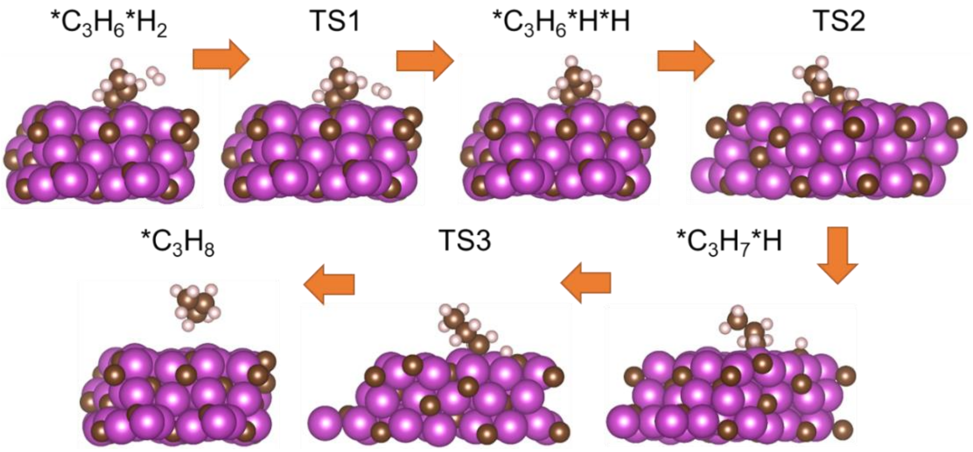

Supplementary Figure 33 | Side-view of propylene hydrogenation reaction coordinate over Fe_5C_2 site. Substrate colors: Fe, pink; C, brown; O, white.

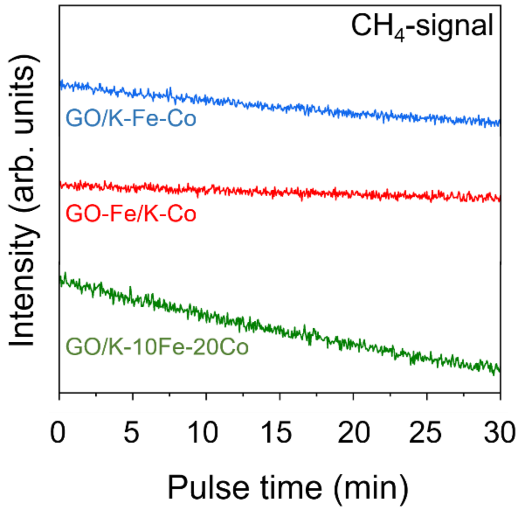

Supplementary Figure 34 | C_3H_6-pulse transient hydrogenation spectra of spent catalysts (CH_4 signal).

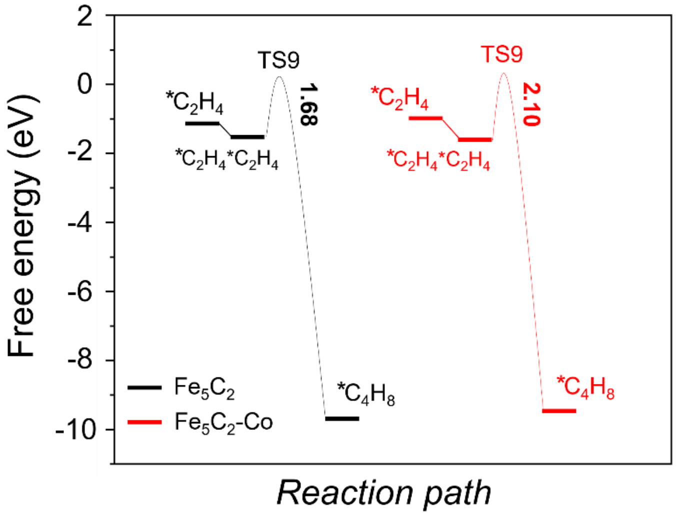

Supplementary Figure 35 | Free energy barriers of ethylene coupled to butene over Fe_5C_2 and Fe_5C_2-Co site.

Note: Over the Fe_5C_2 site, the free energy barrier of ethylene coupling (1.68 eV) was higher than that of C_2H_4 growth to C_3H_6 (1.58 eV), while over the Fe_5C_2-Co site, the free energy barrier of ethylene coupling (2.10 eV) was higher than that of C_2H_4 hydrogenation to C_2H_6 (0.39 eV), indicating that the ethylene coupling pathway was not the ideal path over the dual active sites.

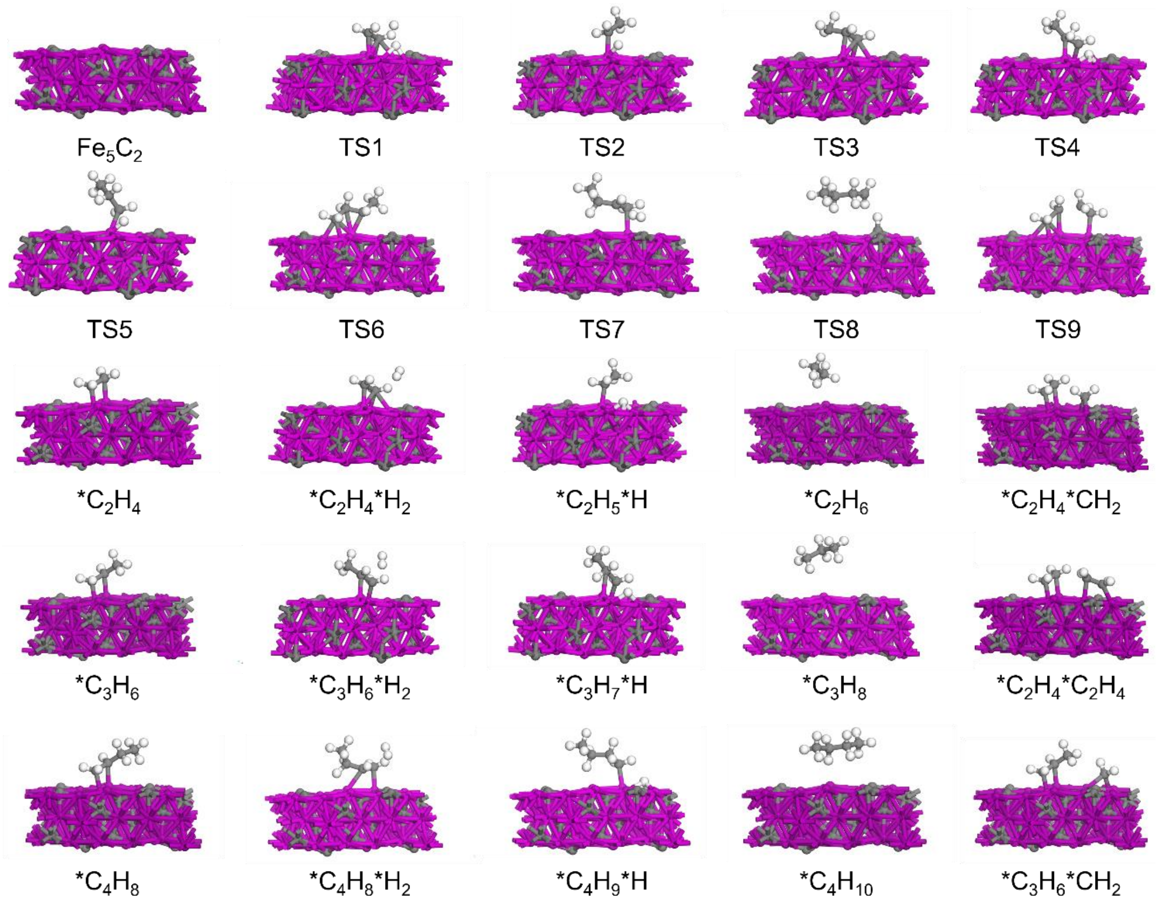

Supplementary Figure 36 | Optimized transition states and intermediates of various steps in carbon chain growth and olefin secondary hydrogenation over Fe_5C_2 sites.

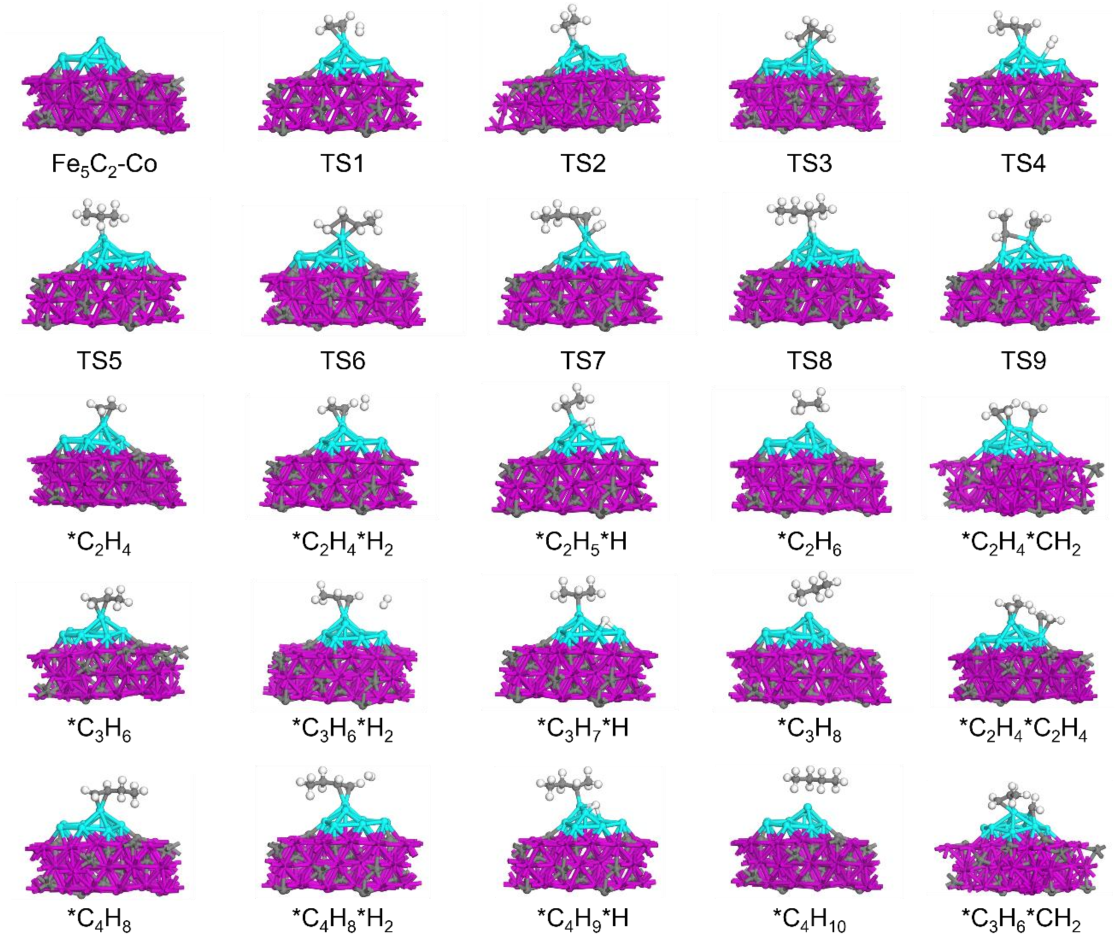

Supplementary Figure 37 | Optimized transition states and intermediates of various steps in carbon chain growth and olefin secondary hydrogenation over Fe_5C_2-Co sites.

Supplementary Tables

Supplementary Table 1 | K, Fe, and Co contents obtained by ICP-OES analysis.

Sample	Elements contents (wt%)		
	K	Fe	Co
GO-Co/K-Fe	1.07	19.33	3.79
GO/K-Fe-Co	1.06	22.75	4.05
GO-Fe-Co/K	0.95	18.84	3.81
GO-Fe/K-Co	1.03	21.19	3.75
GO/K-Fe	1.14	18.91	-
GO-Fe/K	1.03	19.29	-
GO-25Fe/K-Co	1.11	23.83	4.23
GO-30Fe/K-Co	1.01	29.21	3.96

Supplementary Table 2 | Layer distances obtained by Bragg's Law and BET surface areas of GO and rGO.

Sample	Peak (110) 2θ (degree)	d (nm)	S_{BET} (cm^2/g)
GO	8.2	1.1	379.6
rGO	26.3	0.3	44.9

Supplementary Table 3 | Surface element content and Fe/Co ratios obtained by XPS analysis.

Sample	Elements contents (wt%)						Fe/Co
	K 2*p*	Fe 2*p*	Co 2*p*	C 1*s*	O 1*s*	N 1*s*	
GO-Co/K-Fe	0.78	12.25	5.80	62.02	15.53	3.62	2.1
GO/K-Fe-Co	0.52	11.71	6.26	63.12	15.86	2.53	1.9
GO-Fe-Co/K	0.47	3.38	1.97	77.90	14.61	1.67	1.7
GO-Fe/K-Co	0.51	4.07	6.50	72.04	13.98	2.90	0.6
rGO-Fe/K-Co	0.81	11.22	6.56	64.93	14.32	2.16	1.7
GO/K-10Fe-20Co	0.65	8.65	13.13	61.33	13.61	2.63	0.7
GO/K-Fe	1.58	15.30	-	64.84	15.88	2.40	-
GO-Fe/K	1.15	8.24	-	69.12	16.56	4.93	-

Supplementary Table 4 | Surface element content and Fe/Co ratios obtained by SEM mapping analysis.

Sample	Elements contents (wt%)					Fe/Co
	K	Fe	Co	C	O	
GO-Co/K-Fe	0.76	11.27	1.58	60.18	26.21	7.13
GO/K-Fe-Co	0.67	4.01	0.85	77.95	16.52	5.99
GO-Fe-Co/K	0.55	24.15	4.46	45.65	25.19	5.41
GO-Fe/K-Co	1.37	7.38	9.01	53.25	28.99	0.82

Supplementary Table 5 | Element content and Fe/Co ratios of the cross-section obtained by FIB-SEM mapping analysis.

Sample	Elements contents (wt%)				Fe/Co
	Fe	Co	C	O	
GO-Fe/K-Co	7.83	0.95	83.41	7.81	8.42

Supplementary Table 6 | ^{57}Fe Mössbauer parameters of the fresh graphene-supported Fe-Co catalysts.

Sample	Phase ascription	IS (mm s^{-1})	QS (mm s^{-1})	H (T)	Γ (mm s^{-1})	A (%)
GO-Co/K-Fe	Fe^{2+} / Fe^{3+} spm	0.30	0.85	-	0.88	20.20
	Fe_3O_4 (A)	0.65	0.01	40.79	0.50	9.90
	Fe_3O_4 (B)	0.25	-0.01	50.34	0.88	69.90
GO/K-Fe-Co	Fe^{2+} / Fe^{3+} spm	0.30	0.85	-	0.88	3.10
	Fe_3O_4 (A)	0.50	0.01	43.11	0.50	6.40
	Fe_3O_4 (B)	0.25	-0.01	51.28	0.88	90.50
GO-Fe-Co/K	Fe^{2+} / Fe^{3+} spm	0.30	0.85	-	0.88	14.50
	Fe_3O_4 (A)	0.50	0.01	44.16	0.50	10.20
	Fe_3O_4 (B)	0.25	-0.01	50.58	0.88	75.30
GO-Fe/K-Co	Fe^{2+} / Fe^{3+} spm	0.30	0.85	-	0.88	8.10
	Fe_3O_4 (A)	0.50	-0.01	47.36	0.50	13.00
	Fe_3O_4 (B)	0.25	-0.01	52.50	0.88	78.90

Supplementary Table 7 | Co K-edge EXAFS curve fitting parameters of the fresh catalysts.

Sample	Path	CN	R(Å)	σ^2($\times10^{-3}$Å^2)	ΔE_0(eV)	R factor
Co foil	Co-Co	12	2.49	6.2	7.5	0.0002
GO-Co/K-Fe	Co-O	3.8	2.05	6.4	-1.5	0.002
	Co-Co	7.9	3.0	14.7	-1.5	
GO/K-Fe-Co	Co-O	5.3	2.09	6.4	4.6	0.016
	Co-Co	9.6	3.0	12.4	3.2	
GO-Fe-Co/K	Co-O	4.5	2.08	10.2	1.0	0.017
	Co-Co	9.6	2.96	14.7	-6	
GO-Fe/K-Co	Co-O	3.4	2.09	5.4	2.6	0.013
	Co-Co	9.6	2.98	10.8	-1.3	

C*N*: coordination numbers; *R*: bond distance; σ^2: Debye-Waller factors; ΔE_0: the inner potential correction. *R* factor: goodness of fit. Error bounds (accuracies) that characterize the structural parameters obtained by EXAFS spectroscopy were estimated as N ± 20%; R ± 1%; σ^2 ± 20%; ΔE_0 ± 20%. S_0^2 was set as 0.8 for Co-Co, which was obtained from the experimental EXAFS fit of the Co foil reference by fixing CN as the known crystallographic value and was fixed to all the samples.

Supplementary Table 8 | ^{57}Fe Mössbauer parameters of the spent graphene-supported Fe-Co catalysts.

Sample	Phase ascription	IS (mm s^{-1})	QS (mm s^{-1})	H (T)	Γ (mm s^{-1})	A (%)
GO-Co/K-Fe	Fe^{2+} spm	0.25	0.94	-	0.63	18.61
	Fe^{3+} spm	0.16	2.20	-	0.63	6.80
	Fe-C spm	0.15	3.73	-	0.63	8.88
	Fe	0.40	-0.80	34.67	0.75	6.45
	Fe_5C_2 (A)	0.22	0	21.40	0.51	29.89
	Fe_5C_2 (B)	0.16	0	18.54	0.52	29.37
GO/K-Fe-Co	Fe^{2+} spm	0.20	0.92	-	0.53	11.28
	Fe^{3+} spm	0.23	2.17	-	0.53	6.65
	Fe-C spm	0.16	3.59	-	0.53	10.07
	Fe_5C_2 (A)	0.24	0	21.39	0.57	41.82
	Fe_5C_2 (B)	0.19	0	18.50	0.54	30.18
GO-Fe-Co/K	Fe^{2+} spm	0.31	0.97	-	0.69	30.02
	Fe^{3+} spm	0.28	2.39	-	0.69	6.00
	Fe-C spm	0.21	3.64	-	0.69	7.28
	Fe	0.12	-0.15	35.74	0.72	12.12
	Fe_5C_2 (A)	0.21	0	21.63	0.52	26.51
	Fe_5C_2 (B)	0.18	0	18.43	0.66	18.07
GO-Fe/K-Co	Fe^{2+} spm	0.30	0.90	-	0.60	16.22
	Fe^{3+} spm	0.53	2.84	-	0.60	3.94
	Fe-C spm	0.16	3.55	-	0.60	5.49
	Fe	0.04	-0.07	34.00	0.53	13.20
	Fe_5C_2 (A)	0.25	0	21.50	0.38	18.38
	Fe_5C_2 (B)	0.19	0	18.49	0.50	23.98
	Fe_3O_4 (A)	0.63	-0.06	45.34	0.57	8.95
	Fe_3O_4 (B)	0.30	-0.03	48.83	0.39	9.84

Supplementary Table 9 | Co K-edge EXAFS curve fitting parameters of the spent catalysts.

Sample	Path	CN	R(Å)	σ^2(×10^{-3}Å^2)	ΔE_0(eV)	R factor
Co foil	Co-Co	12	2.49	6.2	7.5	0.0002
GO-Co/K-Fe	Co-Co	11.7	2.49	6.2	2.16	0.0029
GO/K-Fe-Co	Co-Co	11.7	2.49	6.3	0.58	0.0033
GO-Fe-Co/K	Co-Co	11.8	2.49	6.3	0.56	0.0034
GO-Fe/K-Co	Co-Co	11.8	2.49	6.3	9.0	0.0009

C*N*: coordination numbers; *R*: bond distance; σ^2: Debye-Waller factors; ΔE_0: the inner potential correction. *R* factor: goodness of fit. Error bounds (accuracies) that characterize the structural parameters obtained by EXAFS spectroscopy were estimated as N ± 20%; R ± 1%; σ^2 ± 20%; ΔE_0 ± 20%. S_0^2 was set as 0.8 for Co-Co, which was obtained from the experimental EXAFS fit of the Co foil reference by fixing CN as the known crystallographic value and was fixed to all the samples.

Supplementary Table 10 | Element contents of the fresh GO-Co/K-Fe obtained by TEM mapping analysis.

Element	Line Type	k factor	Absorption Correction	wt%
C	K series	2.50675	1.00	51.22
K	K series	0.96973	1.00	1.19
Fe	K series	1.19079	1.00	38.90
Co	K series	1.26119	1.00	8.69

Supplementary Table 11 | Element contents of spent catalysts obtained by TEM mapping analysis.

Sample	C (wt%)	Fe (wt%)	Co (wt%)	K (wt%)
GO-Co/K-Fe	95.9	1.6	1.8	0.7
GO/K-Fe-Co	56.4	39.6	2.9	1.1
GO-Fe-Co/K	82.0	14.2	3.3	0.5
GO-Fe/K-Co	59.4	35.7	4.4	0.5

Supplementary References

1. Kresse, G. & Furthmüller, J. Efficiency of Ab-Initio Total Energy Calculations for Metals and Semiconductors Using a Plane-Wave Basis Set. *Comp. Mater. Sci.* **6**, 15–20 (1996).

2. Kresse, G. & Furthmüller, J. Efficient Iterative Schemes for Ab Initio Total-Energy Calculations Using a Plane-Wave Basis Set. *Phys. Rev. B.* **54**, 11169 (1996).

3. Perdew, J., Burke, K. & Ernzerhof, M. Generalized Gradient Approximation Made Simple. *Phys. Rev. Lett.* **78**, 1396 (1997).

4. Kresse, G. & Joubert, D. From Ultrasoft Pseudopotentials to the Projector Augmented-Wave Method. *Phys. Rev. B.* **59**, 1758 (1999).

5. Blöchl, P. Projector Augmented-Wave Method. *Phys. Rev.* B. **50**, 17953 (1994).

6. Crimme, S. et al. A consistent and accurate ab initio parametrization of density functional dispersion correction (DFT-D) for the 94 elements H-Pu. *J. Chem. Phys.* **132**, 154104 (2010).

7. Li, H. et al. A well-defined core-shell-structured capsule catalyst for direct conversion of CO_2 into liquefied petroleum gas. ChemSusChem **13**, 2060–2065 (2020).

8. Fujiwara, M. et al. Synthesis of C_{2+} hydrocarbons by CO_2 hydrogenation over the composite catalyst of Cu–Zn–Al oxide and HB zeolite using two-stage reactor system under low pressure. *Catal. Today* **242**, 255–260 (2015).

9. Wang, S. et al. Highly selective hydrogenation of CO_2 to propane over $GaZrO_x$/H-SSZ-13 composite. *Nat. Catal.* **5**, 1038–1050 (2022).

10. Li, C., Yuan, X. & Fujimoto, K. Direct synthesis of LPG from carbon dioxide over hybrid catalysts comprising modified methanol synthesis catalyst and β-type zeolite. *Appl. Catal. A.* **475**, 155–160 (2014).

11. Natakaranakul, J. et al. Direct synthesis of liquefied petroleum gas from carbon dioxide using a copper/zinc oxide/zirconia/alumina and HY zeolite hybrid catalyst. *ChemistrySelect* **6**, 7103–

7110 (2021).

12. Lu, S. et al. Highly selective synthesis of LPG from CO_2 hydrogenation over In_2O_3/SSZ-13 bifunctional catalyst. *J. Fuel. Chem. Technol.* **49**, 1132–1139 (2021).
